# Supplementary material for: Inference of Breed Structure in Farm Animals: Empirical Comparison between SNP and Microsatellite Performance
Source: Genes (Basel). 2020 Jan 4;11(1):57. doi: 10.3390/genes11010057 (PMC7016564; doi:10.3390/genes11010057)
Supplement: Supplementary file 1 [file genes-11-00057-s001.zip › SupplementaryFiles-R3-652906/TableS1.docx]

| French Sheep (code in brackets) | Micro. dataset from [1]: nb. of indiv. | SNP dataset from [2]: nb. of indiv. |
| --- | --- | --- |
| Berrichon du Cher (BER) | 25 (40) | 19 |
| Blanche du Massif Central (BMC) | 25 (40) | 20 |
| Causses du Lot (CDL) | 25 (40) | 20 |
| Charmoise (CHR) | 25 (30) | 23 |
| Lacaune Lait (LAC) | 25 (80) | 25 (36) |
| Lacaune Viande (LAM) | 25 (80) | 25 (34) |
| Limousine (LIM) | 25 (34) | 18 |
| Manech Tête Rousse (MTR) | 25 (40) | 25 |
| Mérinos de Rambouillet (RAM) | 25 (40) | 27 |
| Mourerous (MOU) | 25 (35) | 16 |
| Noire du Velay (NVE) | 25 (45) | 19 |
| Préalpes du Sud (PAS) | 25 (40) | 17 |
| Rava (RAV) | 25 (39) | 20 |
| Roussin de la Hague (ROU) | 25 (55) | 21 |
| Romane (RMN) | 25 (38) | 19 |
| Rouge de l'Ouest (RWE) | 25 (47) | 17 |
| Tarasconnaise (TAR) | 25 (32) | 15 |
| Total | 425 (755) | 346 (366) |
| Algerian Sheep (code in brackets) | Micro. dataset from [3]: nb. of indiv. | SNP dataset from [4-5]: nb. of indiv. |
| Hamra from Pilot Farms (HAM) | 25 (30) | 9 |
| Ouled Djellal (OD) | 25 (30) | 6 |
| Rembi (REM) | 25 (27) | 6 |
| Sidaou (SID) | 25 (28) | 10 (39) |
| D'men (DMEN) | 13 | 5 |
| Total | 113 (128) | 36 |
| French Cattle (code in brackets) | Micro. dataset from [6]: nb. of indiv. | SNP dataset from [7]: nb. of indiv. |
| Aubrac (AUB) | 25 (50) | 22 |
| Bretonne Pie Noire (BPN) | 25 (31) | 18 |
| Charolais (CHAR) | 25 (55) | 20 |
| Gascon (GASC) | 25 (50) | 22 |
| Rouge des Près (RdP)# | 25 (49) | 23 |
| Montbéliard (MONT) | 25 (30) | 25 (30) |
| Salers (SAL) | 25 (50) | 22 |
| Total | 175 (315) | 152 (157) |

nb.: number, indiv.: individuals, micro.: microsatellites, the number in brackets refers to the number of individuals available in the intial dataset, #: the former name of this breed is Maine Anjou.

REFERENCES

1. Leroy, G.; Danchin-Burge, C.; Palhière, I.; SanCristobal, M.; Nédélec, Y.; Verrier, E.; et al. How do introgression events shape the partitioning of diversity among breeds: a case study in sheep. *Genet Sel Evol.* 2015; 47: 48. doi:10.1186/s12711-015-0131-7

2. Moreno-Romieux, C.; Tortereau, F.; Raoul, J.; Servin, B. High density genotypes of French Sheep populations [Internet]. *Zenodo*; 2017. doi:10.5281/zenodo.237116

3. Gaouar, S.B.S.; Da Silva, A.; Ciani, E.; Kdidi, S.; Aouissat, M.; Dhimi, L.; et al. Admixture and Local Breed Marginalization Threaten Algerian Sheep Diversity. *PLoS One*. 2015; 10. doi:10.1371/journal.pone.0122667

4. Gaouar, S.B.S.; Lafri, M.; Djaout, A.; El-Bouyahiaoui, R.; Bouri, A.; Bouchatal, A.; et al. Genome-wide analysis highlights genetic dilution in Algerian sheep. *Heredity* (Edinb). 2017; 118: 293–301. doi:10.1038/hdy.2016.86

5. Belabdi, I.; Ouhrouch, A.; Lafri, M.; Gaouar, S. B. S.; Ciani, E.; Benali, A. R.; et al. Genetic homogenization of indigenous sheep breeds in Northwest Africa. *Scientific Reports*. 2019; 9(1), 1–13. doi: 10.1038/s41598-019-44137-y

6. Laloë, D.; Jombart, T.; Dufour, A.-B.; Moazami-Goudarzi, K. Consensus genetic structuring and typological value of markers using multiple co-inertia analysis. *Genet Sel Evol.* 2007; 39: 545–567. doi:10.1051/gse:2007021

7. Sempéré, G.; Moazami-Goudarzi, K.; Eggen, A.; Laloë, D.; Gautier, M.; Flori, L. WIDDE: a Web-Interfaced next generation database for genetic diversity exploration, with a first application in cattle. *BMC Genomics*. 2015;16: 940. doi:10.1186/s12864-015-2181-1
