# Supplementary material for: Inference of Breed Structure in Farm Animals: Empirical Comparison between SNP and Microsatellite Performance
Source: Genes (Basel). 2020 Jan 4;11(1):57. doi: 10.3390/genes11010057 (PMC7016564; doi:10.3390/genes11010057)
Supplement: Supplementary file 1 [file genes-11-00057-s001.zip › SupplementaryFiles-R3-652906/TableS2-R3.docx]

| Microsatellites used in the Algerian sheep dataset | Microsatellites used in the French sheep dataset | Microsatellites used in the French cattle dataset |
| --- | --- | --- |
| **BM1824** | **BM8125** | BM1818 |
| **BM8125** | **CSRD247** | **BM1824** |
| **CSRD247** | **HSC** | BM2113 |
| CSSM66 | **HUJ616** | CSRM60 |
| DYMS1 | **ILST11** | **CSSM66** |
| **HSC** | **ILSTS5** | ETH10 |
| **HUJ616** | **INRA49** | ETH152 |
| **ILST11** | **MAF214** | ETH185 |
| **ILSTS5** | **MAF209** | ETH225 |
| INRA35 | **MAF65** | ETH3 |
| **INRA63** | MAF70 | HAUT24 |
| **INRA49** | **MCM42** | HAUT27 |
| **MAF209** | **MCM527** | HEL13 |
| **MAF214** | **OARCP34** | HEL13 |
| MAF33 | **OARFCB128** | HEL5 |
| **MAF65** | **OARFCB193** | HEL9 |
| MCM140 | **OARFCB20** | **ILSTS5** |
| **MCM42** | **OARFCB304** | ILSTS6 |
| **MCM527** | OARJMP29 | INRA23 |
| OARAE129 | **OARJMP58** | INRA32 |
| **OARCP34** | **SRCRSP9** | INRA35 |
| **OARFCB128** |  | INRA37 |
| **OARFCB193** |  | INRA5 |
| **OARFCB20** |  | **INRA63** |
| **OARFCB304** |  | MM12 |
| **OARJMP58** |  | SPS115 |
| **SRCRSP9** |  | **TGLA122** |
| **TGLA122** |  | TGLA126 |
| TGLA53 |  | TGLA227 |
|  |  | TGLA53 |

In bold, microsatellites used in at least two different studies
