# Supplementary material for: Inference of Breed Structure in Farm Animals: Empirical Comparison between SNP and Microsatellite Performance
Source: Genes (Basel). 2020 Jan 4;11(1):57. doi: 10.3390/genes11010057 (PMC7016564; doi:10.3390/genes11010057)
Supplement: Supplementary file 1 [file genes-11-00057-s001.zip › SupplementaryFiles-R3-652906/TableS3.docx]

|  |  | BMC (micro.) | CDL (micro.) | CHR (micro.) | LAC (micro.) | LAM (micro.) | LIM (micro.) | MTR (micro.) | RAM (micro.) | MOU (micro.) | NVE (micro.) | PAS (micro.) | RAV (micro.) | ROU (micro.) | RMN (micro.) | RWE (micro.) | TAR (micro.) |
| --- | --- | --- | --- | --- | --- | --- | --- | --- | --- | --- | --- | --- | --- | --- | --- | --- | --- |
| BER | upper | 0.166 | 0.228 | 0.313 | 0.174 | 0.163 | 0.187 | 0.205 | 0.369 | 0.205 | 0.219 | 0.195 | 0.229 | 0.235 | 0.150 | 0.273 | 0.184 |
|  | lower | 0.085 | 0.108 | 0.139 | 0.091 | 0.093 | 0.086 | 0.112 | 0.176 | 0.110 | 0.093 | 0.100 | 0.099 | 0.138 | 0.069 | 0.139 | 0.100 |
|  | Fst | 0.118 | 0.165 | 0.215 | 0.121 | 0.119 | 0.139 | 0.152 | 0.284 | 0.153 | 0.126 | 0.130 | 0.157 | 0.158 | 0.104 | 0.189 | 0.131 |
| BMC | upper |  | 0.080 | 0.196 | 0.038 | 0.029 | 0.059 | 0.064 | 0.302 | 0.052 | 0.046 | 0.026 | 0.065 | 0.123 | 0.085 | 0.103 | 0.042 |
|  | lower |  | 0.035 | 0.081 | 0.022 | 0.015 | 0.024 | 0.030 | 0.134 | 0.028 | 0.021 | 0.014 | 0.030 | 0.055 | 0.047 | 0.070 | 0.022 |
|  | Fst |  | 0.061 | 0.129 | 0.031 | 0.022 | 0.039 | 0.052 | 0.216 | 0.041 | 0.030 | 0.020 | 0.045 | 0.084 | 0.068 | 0.091 | 0.032 |
| CDL | upper |  |  | 0.242 | 0.087 | 0.075 | 0.110 | 0.133 | 0.323 | 0.106 | 0.089 | 0.088 | 0.088 | 0.146 | 0.156 | 0.124 | 0.084 |
|  | lower |  |  | 0.118 | 0.047 | 0.037 | 0.057 | 0.080 | 0.145 | 0.055 | 0.050 | 0.039 | 0.048 | 0.078 | 0.076 | 0.073 | 0.044 |
|  | Fst |  |  | 0.181 | 0.064 | 0.055 | 0.086 | 0.118 | 0.251 | 0.074 | 0.068 | 0.055 | 0.081 | 0.097 | 0.126 | 0.084 | 0.066 |
| CHR | upper |  |  |  | 0.206 | 0.206 | 0.210 | 0.218 | 0.398 | 0.215 | 0.181 | 0.191 | 0.215 | 0.237 | 0.206 | 0.213 | 0.204 |
|  | lower |  |  |  | 0.093 | 0.099 | 0.098 | 0.100 | 0.210 | 0.098 | 0.087 | 0.093 | 0.094 | 0.128 | 0.109 | 0.112 | 0.106 |
|  | Fst |  |  |  | 0.138 | 0.142 | 0.138 | 0.156 | 0.305 | 0.140 | 0.126 | 0.122 | 0.151 | 0.167 | 0.151 | 0.143 | 0.145 |
| LAC | upper |  |  |  |  | 0.032 | 0.068 | 0.080 | 0.304 | 0.091 | 0.062 | 0.040 | 0.085 | 0.112 | 0.113 | 0.107 | 0.047 |
|  | lower |  |  |  |  | 0.016 | 0.032 | 0.049 | 0.129 | 0.049 | 0.037 | 0.022 | 0.039 | 0.066 | 0.062 | 0.061 | 0.027 |
|  | Fst |  |  |  |  | 0.027 | 0.051 | 0.068 | 0.215 | 0.073 | 0.049 | 0.033 | 0.060 | 0.088 | 0.086 | 0.090 | 0.036 |
| LAM | upper |  |  |  |  |  | 0.060 | 0.072 | 0.278 | 0.073 | 0.046 | 0.035 | 0.074 | 0.105 | 0.099 | 0.093 | 0.038 |
|  | lower |  |  |  |  |  | 0.031 | 0.045 | 0.134 | 0.043 | 0.028 | 0.017 | 0.032 | 0.058 | 0.063 | 0.051 | 0.019 |
|  | Fst |  |  |  |  |  | 0.046 | 0.064 | 0.199 | 0.060 | 0.035 | 0.028 | 0.053 | 0.079 | 0.082 | 0.079 | 0.028 |
| LIM | upper |  |  |  |  |  |  | 0.090 | 0.299 | 0.078 | 0.087 | 0.069 | 0.073 | 0.127 | 0.100 | 0.126 | 0.050 |
|  | lower |  |  |  |  |  |  | 0.047 | 0.133 | 0.044 | 0.036 | 0.034 | 0.041 | 0.071 | 0.058 | 0.073 | 0.029 |
|  | Fst |  |  |  |  |  |  | 0.068 | 0.213 | 0.061 | 0.044 | 0.050 | 0.045 | 0.092 | 0.079 | 0.103 | 0.038 |
| MTR | upper |  |  |  |  |  |  |  | 0.295 | 0.084 | 0.079 | 0.083 | 0.099 | 0.162 | 0.106 | 0.158 | 0.070 |
|  | lower |  |  |  |  |  |  |  | 0.151 | 0.045 | 0.045 | 0.048 | 0.061 | 0.089 | 0.060 | 0.094 | 0.027 |
|  | Fst |  |  |  |  |  |  |  | 0.226 | 0.068 | 0.068 | 0.067 | 0.081 | 0.131 | 0.085 | 0.140 | 0.050 |
| RAM | upper |  |  |  |  |  |  |  |  | 0.314 | 0.284 | 0.296 | 0.316 | 0.327 | 0.257 | 0.323 | 0.274 |
|  | lower |  |  |  |  |  |  |  |  | 0.145 | 0.149 | 0.135 | 0.146 | 0.176 | 0.145 | 0.144 | 0.121 |
|  | Fst |  |  |  |  |  |  |  |  | 0.239 | 0.210 | 0.204 | 0.218 | 0.263 | 0.207 | 0.252 | 0.205 |
| MOU | upper |  |  |  |  |  |  |  |  |  | 0.079 | 0.059 | 0.079 | 0.135 | 0.114 | 0.121 | 0.060 |
|  | lower |  |  |  |  |  |  |  |  |  | 0.043 | 0.033 | 0.044 | 0.068 | 0.068 | 0.060 | 0.033 |
|  | Fst |  |  |  |  |  |  |  |  |  | 0.052 | 0.044 | 0.056 | 0.098 | 0.088 | 0.092 | 0.047 |
| NVE | upper |  |  |  |  |  |  |  |  |  |  | 0.053 | 0.086 | 0.106 | 0.093 | 0.109 | 0.059 |
|  | lower |  |  |  |  |  |  |  |  |  |  | 0.030 | 0.042 | 0.069 | 0.055 | 0.064 | 0.031 |
|  | Fst |  |  |  |  |  |  |  |  |  |  | 0.033 | 0.064 | 0.087 | 0.070 | 0.089 | 0.041 |
| PAS | upper |  |  |  |  |  |  |  |  |  |  |  | 0.080 | 0.123 | 0.095 | 0.096 | 0.047 |
|  | lower |  |  |  |  |  |  |  |  |  |  |  | 0.039 | 0.071 | 0.057 | 0.057 | 0.025 |
|  | Fst |  |  |  |  |  |  |  |  |  |  |  | 0.056 | 0.091 | 0.071 | 0.077 | 0.032 |
| RAV | upper |  |  |  |  |  |  |  |  |  |  |  |  | 0.143 | 0.114 | 0.123 | 0.069 |
|  | lower |  |  |  |  |  |  |  |  |  |  |  |  | 0.083 | 0.068 | 0.067 | 0.037 |
|  | Fst |  |  |  |  |  |  |  |  |  |  |  |  | 0.112 | 0.092 | 0.102 | 0.048 |
| ROU | upper |  |  |  |  |  |  |  |  |  |  |  |  |  | 0.170 | 0.125 | 0.099 |
|  | lower |  |  |  |  |  |  |  |  |  |  |  |  |  | 0.099 | 0.073 | 0.055 |
|  | Fst |  |  |  |  |  |  |  |  |  |  |  |  |  | 0.126 | 0.103 | 0.079 |
| RMN | upper |  |  |  |  |  |  |  |  |  |  |  |  |  |  | 0.172 | 0.101 |
|  | lower |  |  |  |  |  |  |  |  |  |  |  |  |  |  | 0.090 | 0.053 |
|  | Fst |  |  |  |  |  |  |  |  |  |  |  |  |  |  | 0.128 | 0.075 |
| RWE | upper |  |  |  |  |  |  |  |  |  |  |  |  |  |  |  | 0.120 |
|  | lower |  |  |  |  |  |  |  |  |  |  |  |  |  |  |  | 0.058 |
|  | Fst |  |  |  |  |  |  |  |  |  |  |  |  |  |  |  | 0.099 |

|  |  | BMC (SNP) | CDL (SNP) | CHR (SNP) | LAC (SNP) | LAM (SNP) | LIM (SNP) | MTR (SNP) | RAM (SNP) | MOU (SNP) | NVE (SNP) | PAS (SNP) | RAV (SNP) | ROU (SNP) | RMN (SNP) | RWE (SNP) | TAR (SNP) |
| --- | --- | --- | --- | --- | --- | --- | --- | --- | --- | --- | --- | --- | --- | --- | --- | --- | --- |
| BER | upper | 0.136 | 0.176 | 0.183 | 0.151 | 0.139 | 0.171 | 0.163 | 0.326 | 0.132 | 0.147 | 0.139 | 0.152 | 0.166 | 0.088 | 0.133 | 0.145 |
|  | lower | 0.132 | 0.172 | 0.179 | 0.148 | 0.135 | 0.167 | 0.159 | 0.321 | 0.128 | 0.143 | 0.135 | 0.149 | 0.161 | 0.085 | 0.128 | 0.141 |
|  | Fst | 0.134 | 0.174 | 0.181 | 0.150 | 0.137 | 0.169 | 0.161 | 0.323 | 0.130 | 0.145 | 0.137 | 0.151 | 0.163 | 0.087 | 0.130 | 0.143 |
| BMC | upper |  | 0.064 | 0.104 | 0.034 | 0.026 | 0.061 | 0.059 | 0.237 | 0.033 | 0.036 | 0.031 | 0.044 | 0.098 | 0.067 | 0.082 | 0.038 |
|  | lower |  | 0.062 | 0.101 | 0.033 | 0.024 | 0.059 | 0.057 | 0.233 | 0.033 | 0.035 | 0.030 | 0.042 | 0.094 | 0.065 | 0.079 | 0.036 |
|  | Fst |  | 0.063 | 0.102 | 0.033 | 0.025 | 0.060 | 0.058 | 0.235 | 0.033 | 0.035 | 0.030 | 0.043 | 0.096 | 0.066 | 0.081 | 0.037 |
| CDL | upper |  |  | 0.140 | 0.076 | 0.069 | 0.097 | 0.094 | 0.274 | 0.071 | 0.070 | 0.069 | 0.075 | 0.135 | 0.107 | 0.119 | 0.075 |
|  | lower |  |  | 0.137 | 0.074 | 0.066 | 0.094 | 0.091 | 0.268 | 0.068 | 0.068 | 0.066 | 0.072 | 0.131 | 0.104 | 0.115 | 0.072 |
|  | Fst |  |  | 0.139 | 0.075 | 0.067 | 0.096 | 0.093 | 0.271 | 0.070 | 0.069 | 0.068 | 0.074 | 0.133 | 0.105 | 0.117 | 0.073 |
| CHR | upper |  |  |  | 0.115 | 0.107 | 0.133 | 0.128 | 0.299 | 0.105 | 0.111 | 0.105 | 0.116 | 0.145 | 0.121 | 0.130 | 0.111 |
|  | lower |  |  |  | 0.111 | 0.104 | 0.129 | 0.124 | 0.294 | 0.102 | 0.108 | 0.101 | 0.113 | 0.141 | 0.117 | 0.127 | 0.108 |
|  | Fst |  |  |  | 0.113 | 0.105 | 0.131 | 0.126 | 0.297 | 0.104 | 0.110 | 0.103 | 0.114 | 0.143 | 0.119 | 0.128 | 0.109 |
| LAC | upper |  |  |  |  | 0.024 | 0.072 | 0.069 | 0.241 | 0.044 | 0.048 | 0.044 | 0.055 | 0.108 | 0.081 | 0.095 | 0.047 |
|  | lower |  |  |  |  | 0.023 | 0.070 | 0.066 | 0.236 | 0.043 | 0.046 | 0.042 | 0.053 | 0.106 | 0.078 | 0.092 | 0.045 |
|  | Fst |  |  |  |  | 0.024 | 0.071 | 0.068 | 0.239 | 0.044 | 0.047 | 0.043 | 0.054 | 0.107 | 0.079 | 0.094 | 0.046 |
| LAM | upper |  |  |  |  |  | 0.064 | 0.061 | 0.234 | 0.036 | 0.040 | 0.036 | 0.048 | 0.100 | 0.071 | 0.086 | 0.040 |
|  | lower |  |  |  |  |  | 0.062 | 0.060 | 0.229 | 0.036 | 0.038 | 0.034 | 0.046 | 0.097 | 0.068 | 0.083 | 0.038 |
|  | Fst |  |  |  |  |  | 0.063 | 0.060 | 0.232 | 0.036 | 0.039 | 0.035 | 0.047 | 0.099 | 0.069 | 0.085 | 0.039 |
| LIM | upper |  |  |  |  |  |  | 0.087 | 0.270 | 0.065 | 0.068 | 0.065 | 0.070 | 0.127 | 0.098 | 0.112 | 0.068 |
|  | lower |  |  |  |  |  |  | 0.084 | 0.264 | 0.063 | 0.065 | 0.063 | 0.068 | 0.122 | 0.095 | 0.109 | 0.066 |
|  | Fst |  |  |  |  |  |  | 0.086 | 0.267 | 0.064 | 0.066 | 0.064 | 0.069 | 0.124 | 0.096 | 0.111 | 0.067 |
| MTR | upper |  |  |  |  |  |  |  | 0.254 | 0.060 | 0.066 | 0.062 | 0.072 | 0.120 | 0.094 | 0.106 | 0.062 |
|  | lower |  |  |  |  |  |  |  | 0.249 | 0.058 | 0.064 | 0.060 | 0.070 | 0.117 | 0.091 | 0.103 | 0.059 |
|  | Fst |  |  |  |  |  |  |  | 0.251 | 0.059 | 0.065 | 0.061 | 0.071 | 0.118 | 0.093 | 0.105 | 0.061 |
| RAM | upper |  |  |  |  |  |  |  |  | 0.232 | 0.247 | 0.242 | 0.252 | 0.296 | 0.262 | 0.289 | 0.245 |
|  | lower |  |  |  |  |  |  |  |  | 0.232 | 0.242 | 0.238 | 0.247 | 0.290 | 0.257 | 0.283 | 0.240 |
|  | Fst |  |  |  |  |  |  |  |  | 0.232 | 0.245 | 0.240 | 0.250 | 0.293 | 0.259 | 0.286 | 0.243 |
| MOU | upper |  |  |  |  |  |  |  |  |  | 0.041 | 0.032 | 0.050 | 0.095 | 0.061 | 0.082 | 0.037 |
|  | lower |  |  |  |  |  |  |  |  |  | 0.041 | 0.030 | 0.047 | 0.095 | 0.061 | 0.079 | 0.036 |
|  | Fst |  |  |  |  |  |  |  |  |  | 0.041 | 0.031 | 0.049 | 0.095 | 0.061 | 0.080 | 0.036 |
| NVE | upper |  |  |  |  |  |  |  |  |  |  | 0.041 | 0.050 | 0.102 | 0.076 | 0.089 | 0.045 |
|  | lower |  |  |  |  |  |  |  |  |  |  | 0.039 | 0.048 | 0.098 | 0.073 | 0.086 | 0.043 |
|  | Fst |  |  |  |  |  |  |  |  |  |  | 0.040 | 0.049 | 0.100 | 0.074 | 0.088 | 0.044 |
| PAS | upper |  |  |  |  |  |  |  |  |  |  |  | 0.048 | 0.099 | 0.069 | 0.084 | 0.040 |
|  | lower |  |  |  |  |  |  |  |  |  |  |  | 0.046 | 0.096 | 0.066 | 0.081 | 0.038 |
|  | Fst |  |  |  |  |  |  |  |  |  |  |  | 0.047 | 0.098 | 0.067 | 0.082 | 0.039 |
| RAV | upper |  |  |  |  |  |  |  |  |  |  |  |  | 0.109 | 0.082 | 0.093 | 0.053 |
|  | lower |  |  |  |  |  |  |  |  |  |  |  |  | 0.106 | 0.079 | 0.091 | 0.050 |
|  | Fst |  |  |  |  |  |  |  |  |  |  |  |  | 0.107 | 0.080 | 0.092 | 0.051 |
| ROU | upper |  |  |  |  |  |  |  |  |  |  |  |  |  | 0.104 | 0.101 | 0.103 |
|  | lower |  |  |  |  |  |  |  |  |  |  |  |  |  | 0.101 | 0.098 | 0.100 |
|  | Fst |  |  |  |  |  |  |  |  |  |  |  |  |  | 0.103 | 0.100 | 0.102 |
| RMN | upper |  |  |  |  |  |  |  |  |  |  |  |  |  |  | 0.080 | 0.073 |
|  | lower |  |  |  |  |  |  |  |  |  |  |  |  |  |  | 0.077 | 0.070 |
|  | Fst |  |  |  |  |  |  |  |  |  |  |  |  |  |  | 0.078 | 0.071 |
| RWE | upper |  |  |  |  |  |  |  |  |  |  |  |  |  |  |  | 0.090 |
|  | lower |  |  |  |  |  |  |  |  |  |  |  |  |  |  |  | 0.086 |
|  | Fst |  |  |  |  |  |  |  |  |  |  |  |  |  |  |  | 0.088 |

in red the non-overlapping confidence intervals; upper: upper limit of the 95% confidence interval; lower: lower limit of the 95% confidence interval; micro.: microsatellite dataset; SNP: SNP dataset; for breed names see codes in Table S1.
