# Supplementary material for: Inference of Breed Structure in Farm Animals: Empirical Comparison between SNP and Microsatellite Performance
Source: Genes (Basel). 2020 Jan 4;11(1):57. doi: 10.3390/genes11010057 (PMC7016564; doi:10.3390/genes11010057)
Supplement: Supplementary file 1 [file genes-11-00057-s001.zip › SupplementaryFiles-R3-652906/TableS4.docx]

|  | OD (micro.) | REM (micro.) | SID (micro.) | DMEN (micro.) |  |
| --- | --- | --- | --- | --- | --- |
| HAM upper | 0.054 | 0.044 | 0.096 | 0.112 |  |
| lower | 0.026 | 0.023 | 0.049 | 0.056 |  |
| Fst | 0.042 | 0.035 | 0.081 | 0.085 |  |
| OD upper |  | 0.016 | 0.043 | 0.072 |  |
| lower |  | 0.002 | 0.018 | 0.035 |  |
| Fst |  | 0.010 | 0.030 | 0.053 |  |
| REM upper |  |  | 0.041 | 0.074 |  |
| lower |  |  | 0.018 | 0.032 |  |
| Fst |  |  | 0.030 | 0.054 |  |
| SID upper |  |  |  | 0.086 |  |
| lower |  |  |  | 0.038 |  |
| Fst |  |  |  | 0.064 |  |
|  |  |  |  |  |  |
|  | OD (SNP) | REM (SNP) | SID (SNP) | DMEN (SNP) |  |
| HAM upper | 0.055 | 0.053 | 0.083 | 0.088 |  |
| lower | 0.051 | 0.050 | 0.079 | 0.083 |  |
| Fst | 0.053 | 0.051 | 0.081 | 0.086 |  |
| OD upper |  | 0.002 | 0.041 | 0.039 |  |
| lower |  | -0.001 | 0.038 | 0.035 |  |
| Fst |  | 0.001 | 0.040 | 0.037 |  |
| REM upper |  |  | 0.042 | 0.040 |  |
| lower |  |  | 0.038 | 0.037 |  |
| Fst |  |  | 0.040 | 0.039 |  |
| SID upper |  |  |  | 0.052 |  |
| lower |  |  |  | 0.049 |  |
| Fst |  |  |  | 0.050 |  |
|  |  |  |  |  |  |

In red the non-overlapping confidence intervals; upper: upper limit of the 95% confidence interval; lower: lower limit of the 95% confidence interval; micro.: microsatellite dataset; SNP: SNP dataset; for breed names see codes in Table S1.
