# Supplementary material for: Inference of Breed Structure in Farm Animals: Empirical Comparison between SNP and Microsatellite Performance
Source: Genes (Basel). 2020 Jan 4;11(1):57. doi: 10.3390/genes11010057 (PMC7016564; doi:10.3390/genes11010057)
Supplement: Supplementary file 1 [file genes-11-00057-s001.zip › SupplementaryFiles-R3-652906/TableS5.docx]

|  | BPN (micro.) | CHAR (micro.) | GASC (micro.) | RdP (micro.) | MONT (micro.) | SAL (micro.) |
| --- | --- | --- | --- | --- | --- | --- |
| AUB upper | 0.075 | 0.109 | 0.047 | 0.155 | 0.079 | 0.065 |
| lower | 0.041 | 0.042 | 0.025 | 0.087 | 0.031 | 0.020 |
| F_ST_ | 0.061 | 0.075 | 0.037 | 0.125 | 0.062 | 0.040 |
| BPN upper |  | 0.084 | 0.078 | 0.100 | 0.080 | 0.094 |
| lower |  | 0.029 | 0.043 | 0.061 | 0.044 | 0.048 |
| Fst |  | 0.050 | 0.061 | 0.078 | 0.062 | 0.074 |
| CHAR upper |  |  | 0.101 | 0.094 | 0.090 | 0.114 |
| lower |  |  | 0.036 | 0.047 | 0.048 | 0.055 |
| Fst |  |  | 0.067 | 0.069 | 0.066 | 0.085 |
| GASC upper |  |  |  | 0.147 | 0.091 | 0.099 |
| lower |  |  |  | 0.074 | 0.044 | 0.044 |
| Fst |  |  |  | 0.112 | 0.073 | 0.070 |
| RdP upper |  |  |  |  | 0.143 | 0.156 |
| lower |  |  |  |  | 0.093 | 0.085 |
| Fst |  |  |  |  | 0.116 | 0.124 |
| MONT upper |  |  |  |  |  | 0.115 |
| lower |  |  |  |  |  | 0.062 |
| Fst |  |  |  |  |  | 0.095 |
|  |  |  |  |  |  |  |
|  |  |  |  |  |  |  |
|  | BPN(SNP) | CHAR(SNP) | GASC(SNP) | RdP(SNP) | MONT(SNP) | SAL(SNP) |
| AUB upper | 0.072 | 0.048 | 0.042 | 0.101 | 0.082 | 0.048 |
| lower | 0.069 | 0.046 | 0.041 | 0.098 | 0.079 | 0.046 |
| Fst | 0.070 | 0.047 | 0.041 | 0.100 | 0.080 | 0.047 |
| BPN upper |  | 0.060 | 0.070 | 0.079 | 0.100 | 0.093 |
| lower |  | 0.058 | 0.068 | 0.077 | 0.097 | 0.090 |
| Fst |  | 0.059 | 0.069 | 0.078 | 0.098 | 0.091 |
| CHAR upper |  |  | 0.053 | 0.065 | 0.083 | 0.067 |
| lower |  |  | 0.051 | 0.063 | 0.081 | 0.065 |
| Fst |  |  | 0.052 | 0.064 | 0.082 | 0.066 |
| GASC upper |  |  |  | 0.101 | 0.088 | 0.063 |
| lower |  |  |  | 0.098 | 0.085 | 0.061 |
| Fst |  |  |  | 0.100 | 0.087 | 0.062 |
| RdP upper |  |  |  |  | 0.127 | 0.120 |
| lower |  |  |  |  | 0.124 | 0.116 |
| Fst |  |  |  |  | 0.125 | 0.118 |
| MONT upper |  |  |  |  |  | 0.103 |
| lower |  |  |  |  |  | 0.100 |
| Fst |  |  |  |  |  | 0.101 |

In red the non-overlapping confidence intervals; upper: upper limit of the 95% confidence interval; lower: lower limit of the 95% confidence interval; micro.: microsatellite dataset; SNP: SNP dataset; for breed names see codes in Table S1.
